# Advanced imaging reveals enhanced malignancy in glioblastomas involving the subventricular zone: evidence of increased infiltrative growth and perfusion

**DOI:** 10.1007/s11060-024-04849-2

**Published:** 2024-10-10

**Authors:** Michael Griessmair, Severin Schramm, Julian Ziegenfeuter, Julian Canisius, Kirsten Jung, Claire Delbridge, Friederike Schmidt-Graf, Meike Mitsdoerffer, Claus Zimmer, Bernhard Meyer, Marie-Christin Metz, Benedikt Wiestler

**Affiliations:** 1 Dept. of Neuroradiology, Klinikum Rechts der Isar, TU Munich, Ismaningerstr. 22, 81675 Munich, Germany; 2 Dept. of Pathology, TU Munich, 81675 Munich, Germany; 3 Dept. of Neurology, Klinikum Rechts der Isar, TU Munich, 81675 Munich, Germany; 4 Dept. of Neurosurgery, Klinikum Rechts der Isar, TU Munich, 81675 Munich, Germany; 5 TranslaTUM, TU Munich, 81675 Munich, Germany

**Keywords:** Advanced imaging biomarkers, Tumorigenesis of glioblastoma, Fully automated tumor segmentations, 850k methylation analysis

## Abstract

**Background:**

Glioblastoma’s infiltrative growth and heterogeneity are influenced by neural, molecular, genetic, and immunological factors, with the precise origin of these tumors remaining elusive. Neurogenic zones might serve as the tumor stem cells’ nest, with tumors in contact with these zones exhibiting worse outcomes and more aggressive growth patterns. This study aimed to determine if these characteristics are reflected in advanced imaging, specifically diffusion and perfusion data.

**Methods:**

In this monocentric retrospective study, 137 glioblastoma therapy-naive patients (IDH-wildtype, grade 4) with advanced preoperative MRI, including perfusion and diffusion imaging, were analyzed. Tumors and neurogenic zones were automatically segmented. Advanced imaging metrics, including cerebral blood volume (CBV) from perfusion imaging, tissue volume mask (TVM), and free water corrected fractional anisotropy (FA-FWE) from diffusion imaging, were extracted.

**Results:**

SVZ infiltration positively correlated with CBV, indicating higher perfusion in tumors. Significant CBV differences were noted between high and low SVZ infiltration cases at specific percentiles. Negative correlation was observed with TVM and positive correlation with FA-FWE, suggesting more infiltrative tumor growth. Significant differences in TVM and FA-FWE values were found between high and low SVZ infiltration cases.

**Discussion:**

Glioblastomas with SVZ infiltration exhibit distinct imaging characteristics, including higher perfusion and lower cell density per voxel, indicating a more infiltrative growth and higher vascularization. Stem cell-like characteristics in SVZ-infiltrating cells could explain the increased infiltration and aggressive behavior. Understanding these imaging and biological correlations could enhance the understanding of glioblastoma evolution.

**Supplementary Information:**

The online version contains supplementary material available at 10.1007/s11060-024-04849-2.

## Introduction

Despite major research efforts in recent years, the prognosis of glioblastoma patients remains very poor, partly due to diffuse infiltrative growth and intratumoral heterogeneity [1]. Although several neural, molecular, (epi-)genetic and immunological factors are known to drive tumorigenesis, the precise origin of glioblastomas remains unknown [2–4]. However, several studies indicate that there are individual cells within glioblastomas that have stem cell character and are responsible for development and resistance [5].

One hypothesis is that the tumor stem cells (CSC) have their origin in neurogenic zones such as the subventricular zone (SVZ) and the subgranular zone (SGZ) of the dentate gyrus (DG) [6, 7]. It was shown that neural stem cells (NSC) of the SVZ have a similar genetic profile to glioblastoma cells, which could ultimately determine the tumorigenesis of glioblastomas [8]. In addition, glioblastomas close to the SVZ show distinct (epi-)genetic profiles [9]. Clinically, glioblastomas that have contact with the SVZ show a significantly worse outcome and an aggressive, sometimes multifocal growth pattern [10–12]. One study was able to show that irradiation of the ipsilateral SVZ led to longer progression-free survival [13].

Advanced imaging offers an attractive, non-invasive window into tumor biology. Although several radiologic studies have addressed distribution patterns and clinical outcomes [14, 15], data investigating whether SVZ involvement/infiltration affects parameters of advanced MRI imaging, such as perfusion and diffusion parameters, are lacking. Quantitative cerebral blood volume (CBV) derived from perfusion-weighted imaging (PWI) serves as an important MRI biomarker for tumor vascularization as it can detect areas of increased blood volume associated with angiogenesis [16]. For evaluating infiltrative tumor growth, diffusion tensor imaging (DTI) parameters like free water-corrected fractional anisotropy (FA-FWE) and tissue volume masks (TVM) are utilized [17]. These DTI-based metrics can reveal white matter tract disruption and infiltration by tumor cells beyond the visible margins on conventional MRI.

This study thus aims to investigate whether the biology of glioblastomas can be reflected in advanced imaging depending on the SVZ and thus contribute further to the understanding of tumor evolution.

## Methods

### Patient characteristics

A total of 137 patients were included in this retrospective assessment of a prospective monocentric glioma registry. The inclusion criteria were the availability of a preoperative MRI and a detailed postoperative neuropathological diagnosis, including molecular diagnostics, which made a diagnosis according to the fifth WHO classification for CNS tumors 2021 possible [18]. All patients provided informed consent for the prospective glioma registry, which received approval from our local Institutional Review Board (IRB).

### Image analysis

MRI scans were performed using a Philips 3 Tesla whole-body scanner (Achieva or Ingenia models, Philips, Best, The Netherlands). The Philips protocol includes an isotropic FLAIR (voxel size 1 mm3, Echo Time (TE) = 269 ms, Repetition Time (TR) = 4800 ms, Inversion Time (TI) = 1650 ms), isotropic T1w Turbo Field Echo (TFE) (voxel size 1 mm3, TE = 4 ms, TR = 9 ms) before and after contrast, axial T2w (voxel size 0.36 × 0.36 × 4 mm, TE = 87 ms, TR = 3396 ms), DSC perfusion (voxel size 1.75 × 1.75 × 4 mm, TE = 40 ms, TR = 1547 ms, Flip Angle = 75°, 80 dynamics) and DTI (TR/TE: 5,000/78 ms, voxel size of 2 2 2 mm3, 32 diffusion gradient directions, b value 1,000 s/mm2).

All preoperative MRI scans were uniformly processed: Initially, images were co-registered to the SRI24 atlas space [19] using NiftyReg [20] and then skull-stripped with HD-BET [21]. Tumor segmentation was automated using the BraTS Toolkit [22], which was validated in prior studies [23], segmenting tumors into areas of necrosis, contrast-enhancing tumor, and edema/ non-contrast-enhancing tumor. In cases where T2w or FLAIR images were missing, a GAN-based approach was employed to generate the missing sequences to allow automatic segmentation [24]. Resulting segmentations were quality-checked by M.G. ADC and FA maps were derived from DTI using the dipy library [25]. For leakage-corrected CBV calculations, the method by Arzanforoosh et al. was used, which required a mask of normal-appearing white matter for normalization [26]. ANTs Atropos was utilized for this purpose, excluding tumor regions to maintain accuracy [27]. Subsequently, tumor volumes were automatically extracted from segmentation masks. The resulting segmentations were visually quality checked by M.G. and corrected where necessary. Afterwards, summary statistics for ADC and CBV values were compiled.

### Free water correction

Free water correction of preoperative DTI data was conducted using an artificial neural network (ANN) model, as described in a previous study [28]. This ANN was trained with synthetically generated data that included known ground truth, enabling it to learn a nonparametric forward model. This model maps free-water partial volume contamination to volume fractions, effectively decomposing the measured diffusion signal into a “true” diffusion signal and free-water contamination. The model is publicly available at https://github.com/mmromero/dry. Notably, since ADC relies on isotropic diffusion, we only calculated FA maps from free-water corrected DTI data, as well as voxel-wise tissue volume maps, which are essentially given as $\:tvm\:=\:1\:-\:free\:water\:fraction$.

### Automated segmentation of neurogenic zones

**Fig. 1:**
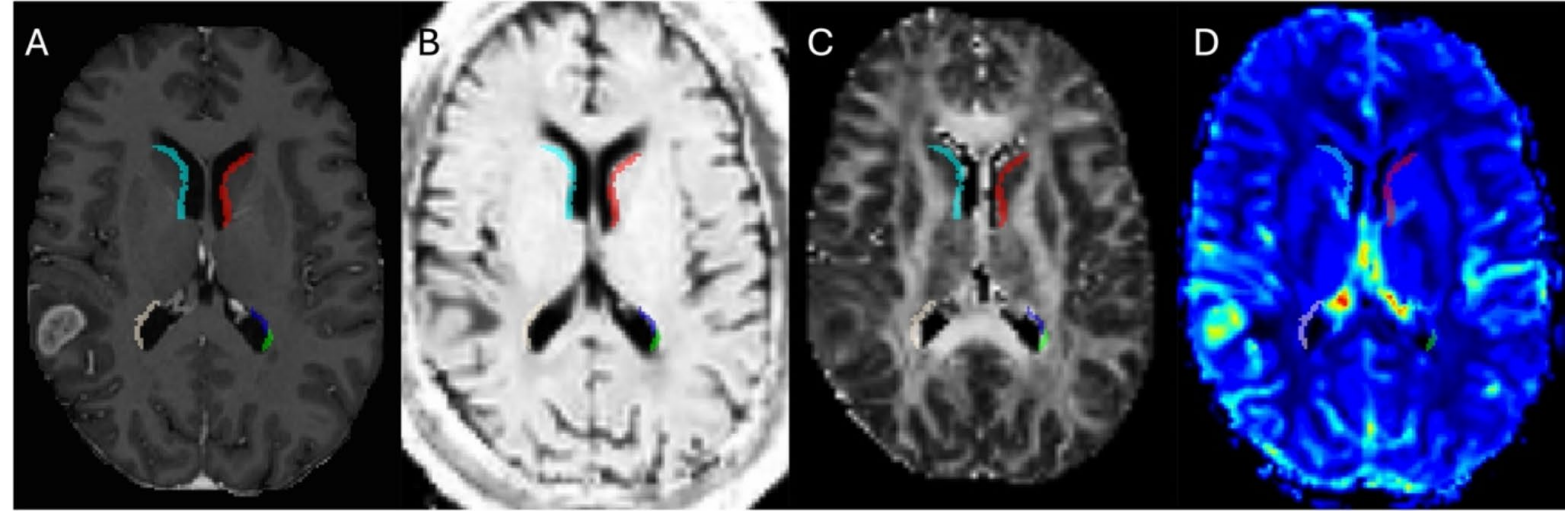
Demonstrates the segmentation of the SVZ; individual subareas of the SVZ are outlined in distinct colors using multiple MRI biomarkers: T1c (A), TVM (B), FA-FWE (C) and CBV (D)

**Fig. 2:**
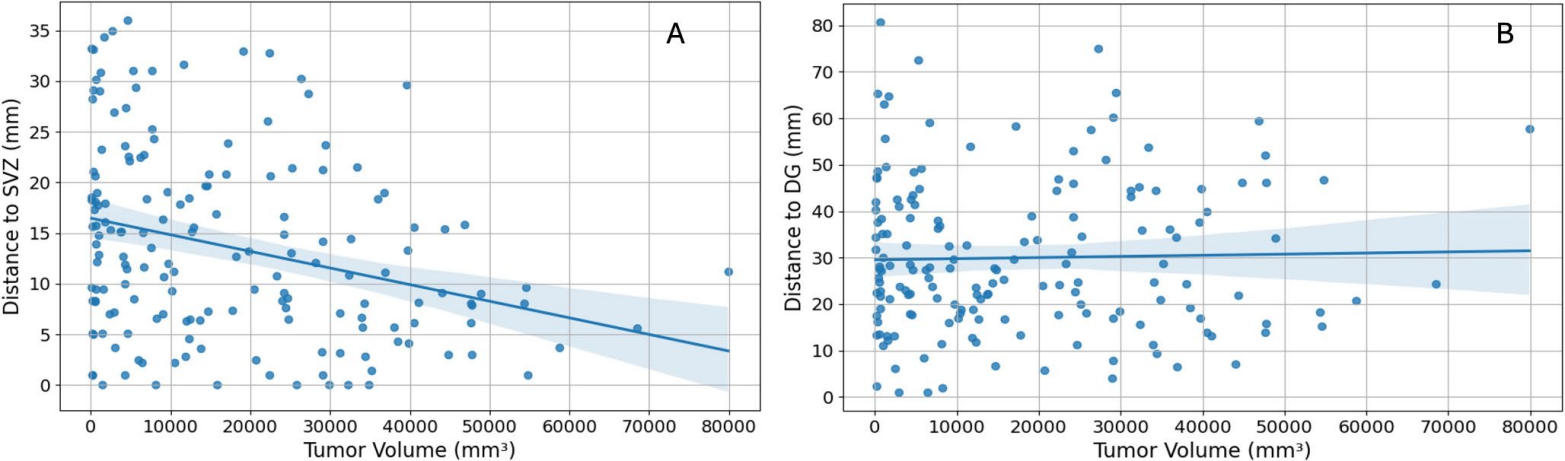
Scatter plot showing the relationship between tumor volume (mm³) and distance to the SVZ (mm) (A) and DG (mm) (B). Panel A: Pearson’s correlation coefficient = -0.30, *p* = 0.000107. Panel B: Pearson’s correlation coefficient = 0.02, *p* = 0.749. Each dot represents a data point. The regression lines indicate the trend, with shaded areas representing the 95% confidence intervals

To automatically segment neurogenic zones in the preoperative MRI, we adopted the methodology developed by Bruil et al. [29] and explained in [10]. In brief, we employed ANTs with parameters established as a robust baseline for the BraTSReg challenge to deformably register the SRI atlas onto each patient’s preoperative MR image. Subsequently, we warped the SVZ atlas from Bruil et al. and the Julich brain atlas for DG segmentation into the patient’s anatomy [29, 30]. All registrations underwent rigorous visual quality checks (by MG) to ensure accuracy. Following the automated tumor and atlas segmentation, we calculated the tumor’s center of mass (of the tumor core, including contrast-enhancing tumor and necrosis) using SciPy like previously described by Jung et al. [10]. The minimum Euclidean distance from this center to the surface of the respective neurogenic zones (SVZ and SGZ) was then automatically determined using SciPy distance functions. This approach ensures accurate and consistent distance calculations, even when dealing with complex structures like the SVZ. This methodology provides a robust framework for quantitatively assessing the spatial relationships between glioblastomas and neurogenic zones. For a detailed visual representation of this process, please refer to Figs. 1 and 2 in our previous publication [10]. The percentage of tumor infiltration is represented by the overlap of segmentation of tumor core and SVZ.

### Neuropathology

Following formalin fixation and paraffin embedding, tissue samples underwent standard neuropathological diagnosis. Beyond traditional histological and immunohistochemical techniques, an 850k methylation analysis was performed on the extracted DNA using the Illumina EPIC 850k Methylation Array BeadChip (Illumina Inc., San Diego, CA, USA). The methylation data was analyzed utilizing the Brain Tumor Classifier from the German Cancer Research Center (DKFZ) and the University of Heidelberg [31, 32]. This comprehensive approach culminated in an integrated diagnosis that combined histology, immunohistochemistry, and molecular pathology, adhering to the guidelines of the 2021 5th edition of the WHO classification [18].

### Statistic and data analysis

All statistical analyses were conducted using Python 3 with Matplotlib, Scipy, Seaborn, and Statsmodels libraries. The study aimed to investigate the relationships between tumor infiltration into the SVZ and various metrics, including TVM, CBV and FA-FWE.

Quantitative metrics were first extracted and normalized to compute percentiles (P5, P25, P50, P75, P95) for TVM, CBV, and FA-FWE. Pearson correlation coefficients were used to assess the relationships between SVZ infiltration and the metrics. Independent T-tests were employed to compare the average values of TVM, CBV, and FA-FWE between groups with different levels of SVZ infiltration. Additionally, Pearson correlation coefficients were computed to evaluate the relationship between the minimum distance from the tumor center of mass to the SVZ and the various metrics. Visualizations with scatter blots were used to illustrate the relationships between SVZ infiltration and the metrics.

## Results

### Patient data

In this study, we focused exclusively on patients diagnosed with grade 4 IDH-wildtype glioblastoma (GBM). The cohort consisted of 137 patients, with an average age of 65 years. Among them, 86 were men, and 51 were women. Multifocal tumor manifestations, defined as the presence of more than one contrast-enhancing lesion, were observed in 30 cases. Perfusion data were available for 117 patients, and diffusion parameters were available for 120 patients. A total of 107 patients had both perfusion and diffusion data. Additionally, we assessed the treatment status of the patients. All patients were preoperative and did not receive any neoadjuvant therapy. Figure 1 illustrating an example for one patient consisting of T1c, TVM, CBV and FA-FWE with segmentation mask for SVZ.

### Tumor volume in relation to distance to neurogenic zones

We examined the relationship between tumor volume and proximity to both the SVZ and DG (see Fig. 2). Our findings reveal a significant negative correlation between tumor volume and distance to SVZ (Pearson’s correlation coefficient = -0.30, *p* = 0.000107) (see Fig. 2A). These results indicate that glioblastomas arising closer to (or even from) the SVZ have a larger tumor volume. To verify that the observed correlation is not simply due to larger tumors having more SVZ infiltration, we also examined the correlation with the DG, another neurogenic zone. The lack of a significant correlation between tumor volume and DG infiltration (Pearson’s correlation coefficient = 0.02, *p* = 0.749) suggests that the association with SVZ is not due to a systematic bias where larger tumors are always closer to neurogenic zones (see Fig. 2B).

### Tumor perfusion in correlation to SVZ distance and infiltration

**Fig. 3:**
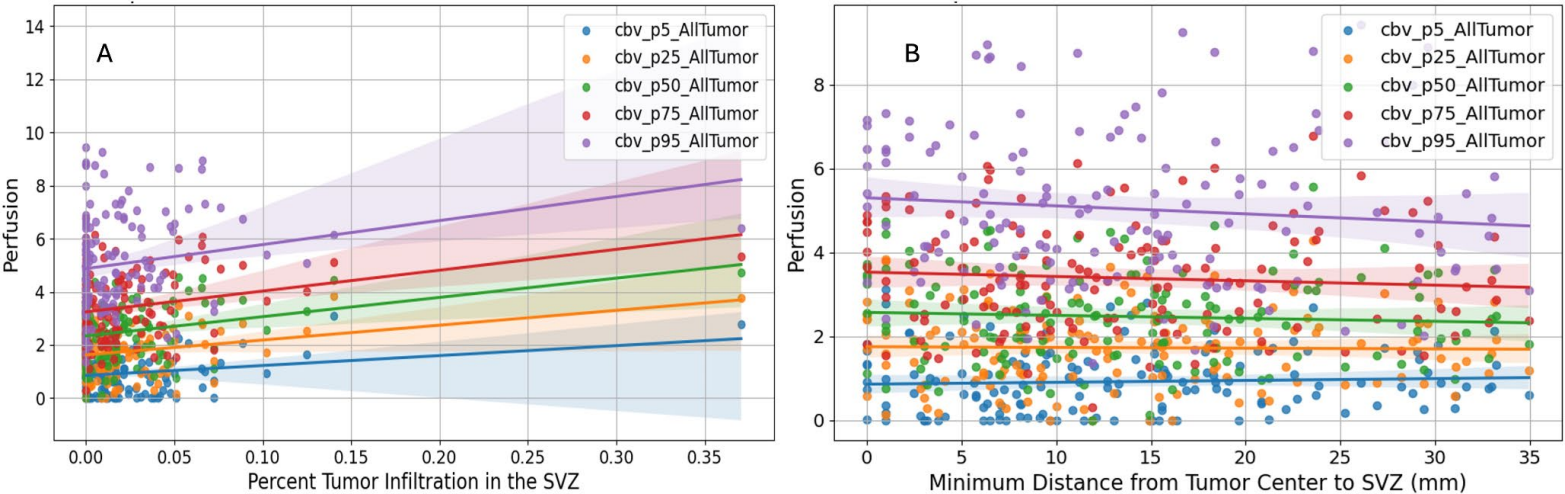
Correlation between tumor perfusion and percent of tumor infiltration in the SVZ (A) and distance to SVZ (B). Panel A shows significant positive correlations between SVZ infiltration and CBV metrics: CBV P5 (*r* = 0.19, *p* = 0.019), CBV P25 (*r* = 0.24, *p* = 0.004), CBV P50 (*r* = 0.26, *p* = 0.001), CBV P75 (*r* = 0.24, *p* = 0.004), and CBV P95 (*r* = 0.19, *p* = 0.021). Panel B shows no significant correlations between tumor distance from the SVZ and perfusion metrics: CBV P5 (*r* = 0.05, *p* = 0.514) and CBV P95 (*r* = -0.10, *p* = 0.248). Each dot represents a data point. The shaded areas represent the 95% confidence intervals

We found significant positive correlations between the extent of tumor infiltration into the SVZ and CBV metrics (see Fig. 3). Specifically, significant correlations were observed across all examined CBV percentiles: CBV P5 (*r* = 0.19, *p* = 0.019), CBV P25 (*r* = 0.24, *p* = 0.004), CBV P50 (*r* = 0.26, *p* = 0.001), CBV P75 (*r* = 0.24, *p* = 0.004), and CBV P95 (*r* = 0.19, *p* = 0.021) (see Table S1). These findings indicate that greater SVZ infiltration is associated with increased perfusion values (see Fig. 3A).

We also CBV values between cases with high (above median) and low (below median) SVZ infiltration. Significant differences were found at CBV P5 and CBV P95, with p-values of 0.004 and 0.048, respectively, suggesting that higher SVZ infiltration correlates with higher perfusion at these percentiles. However, no significant differences were observed at CBV P25, CBV P50, and CBV P75 (see Table S1).

Additionally, we explored the impact of the tumor’s distance from the SVZ on perfusion metrics (see Fig. 3B). The results showed no significant correlations, indicating that the proximity of the tumor to the SVZ does not significantly affect perfusion metrics. The correlation coefficients were consistently weak and not statistically significant, such as for CBV P5 (*r* = 0.05, *p* = 0.514) and CBV P95 (*r* = -0.10, *p* = 0.248) (see Table S2).

### Tissue volume fraction in relation to SVZ distance and infiltration

**Fig. 4:**
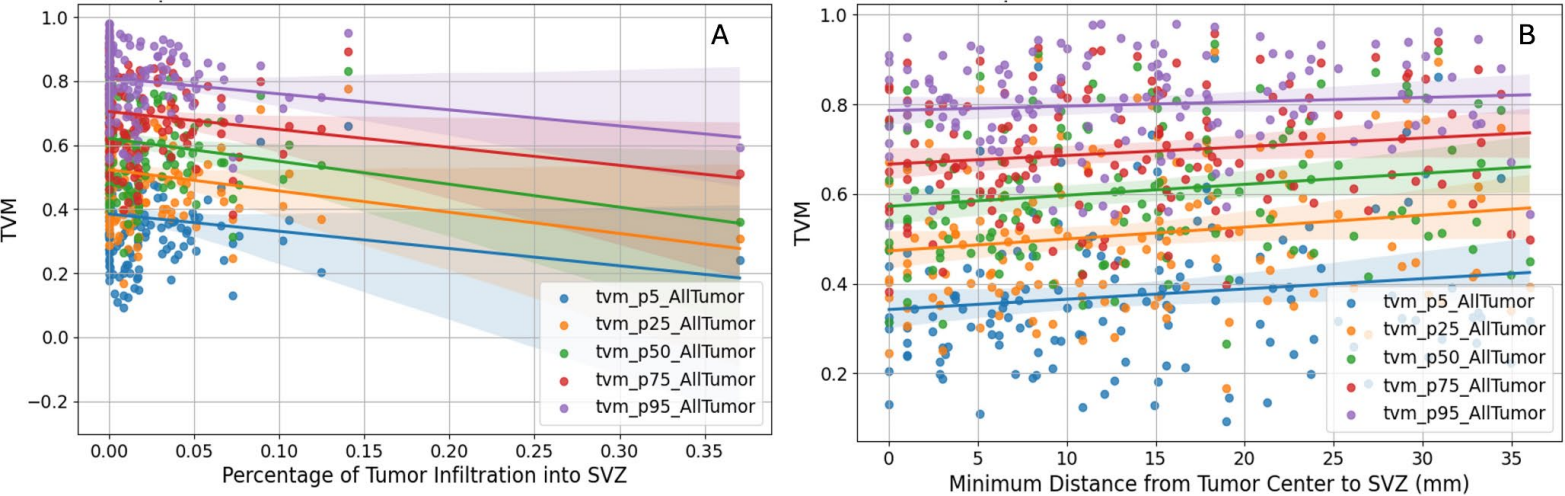
Correlation between TVM and percent of tumor infiltration in the SVZ (A) and distance to SVZ (B). Panel A shows negative correlations between SVZ infiltration and TVM across percentiles: TVM P5 (*r* = -0.13, *p* = 0.109), TVM P25 (*r* = -0.17, *p* = 0.042), TVM P50 (*r* = -0.20, *p* = 0.019), TVM P75 (*r* = -0.17, *p* = 0.042), and TVM P95 (*r* = -0.19, *p* = 0.027). All but TVM P5 were statistically significant (*p* < 0.05). Panel B shows positive correlations between tumor distance from the SVZ and TVM: TVM P5 (*r* = 0.14, *p* = 0.097), TVM P25 (*r* = 0.17, *p* = 0.048), TVM P50 (*r* = 0.16, *p* = 0.052), TVM P75 (*r* = 0.14, *p* = 0.087), and TVM P95 (*r* = 0.09, *p* = 0.300). Only TVM P25 was statistically significant (*p* < 0.05). Each dot represents a data point. The shaded areas represent the 95% confidence intervals

Assessing the correlation between TVM with tumors contacting/infiltrating the SVZ revealed that the infiltration of the SVZ correlates with a more infiltrative phenotype (Fig. 4A). Negative correlations were observed across different TVM percentiles: TVM P5 (*r* = -0.13 *p* = 0.109), TVM P25 (*r*= -0.17 *p* = 0.042), TVM P50 (*r*= -0.20 *p* = 0.019), TVM P75 (*r*= -0.17 *p* = 0.042), and for TVM P95 (*r*= -0.19 *p* = 0.027). Except for TVM p5, all correlations were statistically significant (*p* < 0.05) (see Table S3).

Cases with SVZ infiltration showed lower average TVM values compared to those without infiltration: Independent t-tests confirmed these differences were statistically significant over all percentiles (all *p* < 0.05), highlighting a significant relationship between SVZ infiltration and reduced TVM values (see Table S3).

In addition, correlations were examined between the distance from the tumor center of mass (CoM) to the SVZ and TVM (Fig. 4B). The analysis revealed positive correlations: TVM P50 (*r* = 0.14 *p* = 0.097), TVM P25 (*r* = 0.17 *p* = 0.048), TVM P50 (*r* = 0.16 *p* = 0.052), TVM P75 (*r* = 0.14 *p* = 0.087), and TVM P95 (*r* = 0.09 *p* = 0.300). TVM P25 was the only statistically significant correlation (*p* < 0.05), while others did not reach significance (*p* ≥ 0.05) (see Table S4). Furthermore, comparison of average TVM values between tumors located closer (≤ median distance) and farther (> median distance) from the SVZ showed no statistically significant differences across percentiles (all *p* > 0.05). These findings suggest a modest association between proximity to the SVZ and TVM at specific percentiles but no significant differences in overall TVM values based on distance to the SVZ (see Table S4).

### FA-FWE in relation to SVZ infiltration and distance

**Fig. 5:**
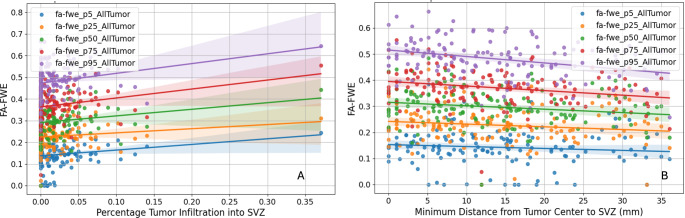
Correlation between FA-FW) and percent of tumor infiltration in the SVZ (A) and distance to SVZ (B). Panel A shows positive correlations between SVZ infiltration and FA-FWE values at different percentiles: P50 (*r* = 0.18, *p* = 0.030), P75 (*r* = 0.18, *p* = 0.003), and P95 (*r* = 0.24, *p* = 0.004), while P5 and P25 were not statistically significant (*p* > 0.05). Panel B shows negative correlations between tumor distance from the SVZ and FA-FWE values: P50 (*r* = -0.18, *p* = 0.023), P75 (*r* = -0.22, *p* = 0.003), and P95 (*r* = -0.24, *p* = 0.001). Each dot represents a data point. The shaded areas represent the 95% confidence intervals

Consistent with the SVZ and TVM correlation described above, the FA-FWE values also showed positive correlations, supporting the hypothesis that SVZ infiltration leads to more infiltrative tumor growth (see Fig. 5). The Pearson correlation analysis revealed varying degrees of positive correlations between the percentage of tumor infiltration into the SVZ and different FA-FWE values. These were significant for P50 (*r* = 0.18 *p* = 0.030), P75 (*r* = 0.18 *p* = 0.003), and P95 (*r* = 0.24 *p* = 0.004), while P5 and P25 did not reach significance (*p* > 0.05). Furthermore, average FA-FWE values were higher in cases with high SVZ (above median) infiltration compared low infiltration (below median). Independent t-tests confirmed that the differences at P75 (*p* = 0.013) and P95 (*p* = 0,002) were statistically significant. However, the differences at P5, P25, and P50 were not statistically significant (*p* > 0.05). The Pearson correlation analysis also revealed varying degrees of negative correlations between the minimum distance from the center of the tumor mass to the SVZ and different FA-FWE values. These were significant for P50 (*p* = 0.023), P75 (*p* = 0.003), and P95 (*p* = 0.001). P5 and P25 did not reach significance (*p* > 0.05). Further analysis compared average FA-FWE values for tumors located close to and far from the SVZ. The t-tests indicated that the differences in FA-FWE values were statistically significant for P25 (*p* = 0.040), P50 (*p* = 0.038), P75 (*p* = 0.023), and P95 (*p* = 0.003). However, the difference for FA-FWE P5 was not statistically significant (*p* > 0.05).

## Discussion

Several works proposed a more aggressive clinical course and infiltrative growth of glioblastoma with contact to the SVZ, a niche of tumor stem cells. However, it is presently unclear how these characteristics of tumors associated with the SVZ are reflected in advanced imaging. In our study, we were able to confirm this malignant, infiltrative biology of SVZ-associated tumors by analyzing various diffusion parameters and perfusion parameters.

To date, molecular genetic differences have been identified between glioblastomas that come into contact with the SVZ and those that do not make contact. Accordingly, Adeberg et al. demonstrated that SVZ + and SVZ- tumors exhibit distinct methylation signatures [9]. In line with the rest of the literature, these SVZ + glioblastomas were associated with a poorer outcome [9, 10]. Furthermore, classic mutations of glioblastomas could be detected in the SVZ of glioblastoma patients, even if the tumor had no contact to the SVZ on imaging [33]. These data could indicate that the SVZ plays a role in tumorigenesis, which leads to more infiltrative growth with a poorer response to therapy.

This study aimed to assess whether the different tumor biology/ infiltrative growth of glioblastomas with and without SVZ infiltration could be reflected in biology/advanced MRI imaging including quantitative analysis of perfusion and diffusion.

Although the results were not all significant, there are indications that infiltration of the SVZ correlates with higher tumor perfusion. This could be due to the increased stemness of the tumors or the genetic profile. One study showed that SVZ + glioblastoma expresses more VEGF, which is in line with our findings. However, it is important to note that this finding was not confirmed in a larger cohort [32], so the relevance of this observation remains uncertain [34].

To evaluate the infiltrative growth of glioblastoma, we utilized various parameters from diffusion imaging. A challenge in this process is the contamination of tumor regions with free water. To address this, we applied the method developed by Molino et al. [28], which was validated in a study by Metz et al. [17]. Our results indicated that TVM values were negatively associated with increasing SVZ infiltration, while FA-FWE values were positively associated. In prior work, Metz et al. showed significantly lower FA-FWE- values in areas of later recurrence, i.e., areas with higher tumor cell density. Both the TVM and FA-FWE data we found here therefore congruently point to a scenario where with increasing contact to the SVZ, tumors tend to be more infiltrative. This is further corroborated by the fact, that these tumors also are larger. In view of the important role the diffuse nature of glioblastoma plays for their poor prognosis, our insights offer a clear link between contact to the SVZ and the poor clinical course of these patients.

Additionally, our analysis revealed that tumors with greater infiltration into the SVZ tend to have larger tumor volumes. To ensure that this observed correlation is not merely a result of larger tumors having more SVZ infiltration, we also investigated the correlation with the dentate gyrus (DG), another neurogenic zone. No significant correlation was found in this case.

In summary, our results indicate that glioblastomas with SVZ infiltration exhibit more infiltrative growth with slightly higher tumor perfusion potentially linked to increased stemness. This could be an explanation for the worse outcome.

### Limitations

Our study has several limitations. Being a monocentric retrospective study, it is not broadly generalizable, although we employed high imaging standards and extended molecular diagnoses. Due to the lack of statistical significance in some analyses, certain aspects remain hypothetical and require further validation. As this was an exploratory study, we did not perform a multi-testing correction. A follow-up study with a larger sample size and a confirmatory design is necessary to reliably validate or refute the relationships and effects observed in this initial exploratory investigation. Nonetheless, the use of fully automated tumor segmentations and a validated workflow for perfusion and DTI data ensured high methodological standards. Our investigation into the involvement of the SVZ in glioblastoma evolution provides interesting insights that could benefit the scientific community.

## Conclusion

The findings from our study suggest that the quantitative analysis of advanced imaging parameters, including free water corrected DTI and perfusion values, can reflect the infiltrative growth patterns of glioblastomas in contact with the SVZ. While these results provide valuable insights into the evolution and potential origins of glioblastoma, further research is necessary to fully understand the role of the SVZ and its progenitor cells in glioblastoma pathophysiology. Our study highlights the potential importance of neurogenic niches in the aggressive behavior of these tumors, but additional studies are needed to confirm these findings and explore their implications for therapeutic strategies.

## Electronic supplementary material

Below is the link to the electronic supplementary material.

Supplementary Material 1

## Data Availability

No datasets were generated or analysed during the current study.
