# Supplementary material for: Advanced imaging reveals enhanced malignancy in glioblastomas involving the subventricular zone: evidence of increased infiltrative growth and perfusion
Source: J Neurooncol. 2024 Oct 10;171(2):343–50. doi: 10.1007/s11060-024-04849-2 (PMC11695386; doi:10.1007/s11060-024-04849-2)
Supplement: Supplementary file 1 — Supplementary Material 1 [file 11060_2024_4849_MOESM1_ESM.docx]

**Supplementary material**

| Percentile | Correlation Coefficient (r) | p-value | Mean Value (High SVZ Infiltration) | Mean Value (Low SVZ Infiltration) | t-value | p-value (t-test) |
| --- | --- | --- | --- | --- | --- | --- |
| CBV P5 | 0.19 | **0.019** | 0.7487 | 1.1005 | -2.93 | **0.004** |
| CBV P25 | 0.24 | **0.004** | 1.6496 | 1.8172 | -1.11 | 0.268 |
| CBV P50 | 0.26 | **0.001** | 2.4741 | 2.4821 | -0.05 | 0.964 |
| CBV P75 | 0.24 | **0.004** | 3.4667 | 3.3190 | 0.69 | 0.490 |
| CBV P95 | 0.19 | **0.021** | 5.3417 | 4.7449 | 2.00 | **0.048** |

*Table S1: Table 1: Correlation between SVZ Infiltration and Perfusion Values (CBV)*

| Percentile | Correlation Coefficient (r) | p-value | Mean Value (Close to SVZ) | Mean Value (Far to SVZ) | t-value | p-value (t-test) |
| --- | --- | --- | --- | --- | --- | --- |
| CBV P5 | 0.05 | 0.514 | 0.842 | 1.006 | -1.34 | 0.184 |
| CBV P25 | -0.02 | 0.836 | 1.695 | 1.772 | -0.51 | 0.612 |
| CBV P50 | -0.06 | 0.469 | 2.483 | 2.473 | 0.05 | 0.958 |
| CBV P75 | -0.07 | 0.377 | 3.414 | 3.372 | 0.19 | 0.846 |
| CBV P95 | -0.10 | 0.248 | 5.118 | 4.972 | 0.48 | 0.631 |

*Table S2: Correlation between Distance to SVZ and Perfusion Values (CBV)*

| Percentile | Correlation Coefficient (r) | p-value | Mean Value (High SVZ Infiltration) | Mean Value (Low SVZ Infiltration) | t-value | p-value (t-test) |
| --- | --- | --- | --- | --- | --- | --- |
| TVM P5 | -0.13 | 0.109 | 0.318 | 0.459 | -2.60 | **0.011** |
| TVM P25 | -0.17 | **0.042** | 0.459 | 0.587 | -2.07 | **0.042** |
| TVM P50 | -0.20 | **0.019** | 0.564 | 0.671 | -2.38 | **0.019** |
| TVM P75 | -0.17 | **0.042** | 0.661 | 0.742 | -2.07 | **0.042** |
| TVM P95 | -0.19 | **0.027** | 0.785 | 0.822 | -2.25 | **0.027** |

*Table S3: Correlation between SVZ Infiltration and Tissue Volume Mask Values (TVM)*

| Percentile | Correlation Coefficient (r) | p-value | Mean Value (Close to SVZ) | Mean Value (Far from SVZ) | t-value | p-value (t-test) |
| --- | --- | --- | --- | --- | --- | --- |
| TVM P5 | 0.14 | 0.097 | 0.318 | 0.459 | -1.66 | 0.089 |
| TVM P25 | 0.17 | **0.048** | 0.459 | 0.587 | -2.01 | 0.081 |
| TVM P50 | 0.16 | 0.052 | 0.564 | 0.671 | -1.95 | 0,107 |
| TVM P75 | 0.14 | 0.087 | 0.661 | 0.742 | -1.71 | 0.120 |
| TVM P95 | 0.09 | 0.300 | 0.785 | 0.822 | -1.04 | 0.248 |

*Table S4: Correlation between Distance to SVZ and Tissue Volume Mask Values (TVM)*

| Percentile | Correlation Coefficient (r) | p-value | Mean Value (High SVZ Infiltration) | Mean Value (Low SVZ Infiltration) | t-value | p-value (t-test) |
| --- | --- | --- | --- | --- | --- | --- |
| FA-FWE P5 | 0.15 | 0.071 | 0.140508 | 0.145687 | -0.45 | 0.650 |
| FA-FWE P25 | 0.11 | 0.173 | 0.231725 | 0.224204 | 0.68 | 0.498 |
| FA-FWE P50 | 0.18 | **0.030** | 0.305593 | 0.289056 | 1.50 | 0.137 |
| FA-FWE P75 | 0.24 | **0.003** | 0.385263 | 0.357440 | 2.53 | **0.013** |
| FA-FWE P95 | 0.24 | **0.004** | 0.501428 | 0.462498 | 3.24 | **0.002** |

*Table S5: Correlation between SVZ Infiltration and FA-FWE Values*

| Percentile | Correlation Coefficient (r) | p-value | Mean Value (Close to SVZ) | Mean Value (Far from SVZ) | t-value | p-value (t-test) |
| --- | --- | --- | --- | --- | --- | --- |
| FA-FWE P5 | -0.10 | 0.215 | 0.1528 | 0.1333 | 1.73 | 0.085 |
| FA-FWE P25 | -0.15 | **0.071** | 0.2392 | 0.2165 | 2.08 | **0.040** |
| FA-FWE P50 | -0.19 | **0.023** | 0.3087 | 0.2857 | 2.10 | **0.038** |
| FA-FWE P75 | -0.25 | **0.003** | 0.3838 | 0.3585 | 2.31 | **0.023** |
| FA-FWE P95 | -0.32 | **<0.001** | 0.5000 | 0.4634 | 2.97 | **0.003** |

*Table S6: Correlation between Distance to SVZ and FA-FWE Values*
